# Machine Learning-Based Surgical Planning for Neurosurgery: Artificial Intelligent Approaches to the Cranium

**DOI:** 10.3389/fsurg.2022.863633

**Published:** 2022-04-29

**Authors:** Tolga Turan Dundar, Ismail Yurtsever, Meltem Kurt Pehlivanoglu, Ugur Yildiz, Aysegul Eker, Mehmet Ali Demir, Ahmet Serdar Mutluer, Recep Tektaş, Mevlude Sila Kazan, Serkan Kitis, Abdulkerim Gokoglu, Ihsan Dogan, Nevcihan Duru

**Affiliations:** ^1^Bezmiâlem Vakif Üniversitesi, Istanbul, Turkey; ^2^Kocaeli University, Izmit, Turkey; ^3^Private System Hospital, Kayseri, Turkey; ^4^Ankara University, Ankara, Turkey; ^5^Kocaeli Health and Technology University, Başiskele, Turkey

**Keywords:** approaches, neurosurgery, neurosurgical planning, machine learning, cranial approaches, artificial intelligence (AI), brain tumor

## Abstract

**Objectives:**

Artificial intelligence (AI) applications in neurosurgery have an increasing momentum as well as the growing number of implementations in the medical literature. In recent years, AI research define a link between neuroscience and AI. It is a connection between knowing and understanding the brain and how to simulate the brain. The machine learning algorithms, as a subset of AI, are able to learn with experiences, perform big data analysis, and fulfill human-like tasks. Intracranial surgical approaches that have been defined, disciplined, and developed in the last century have become more effective with technological developments. We aimed to define individual-safe, intracranial approaches by introducing functional anatomical structures and pathological areas to artificial intelligence.

**Methods:**

Preoperative MR images of patients with deeply located brain tumors were used for planning. Intracranial arteries, veins, and neural tracts are listed and numbered. Voxel values of these selected regions in cranial MR sequences were extracted and labeled. Tumor tissue was segmented as the target. Q-learning algorithm which is a model-free reinforcement learning algorithm was run on labeled voxel values (on optimal paths extracted from the new heuristic-based path planning algorithm), then the algorithm was assigned to list the cortico-tumoral pathways that aim to remove the maximum tumor tissue and in the meantime that functional anatomical tissues will be least affected.

**Results:**

The most suitable cranial entry areas were found with the artificial intelligence algorithm. Cortico-tumoral pathways were revealed using Q-learning from these optimal points.

**Conclusions:**

AI will make a significant contribution to the positive outcomes as its use in both preoperative surgical planning and intraoperative technique equipment assisted neurosurgery, its use increased.

## Introduction

The gold standard surgical strategy for the majority of intra-axial tumors is maximum tumor resection with minimal loss of neurological function (1). The tumoral mass effect on the brain tissue was eliminated while reaching the histological diagnosis with surgical resection. Aggressive surgery also increases radiotherapy and chemotherapy effectiveness in patients who require them by reducing the tumor burden. Subcutaneous tissue incision, craniotomy size, and dura are standardized. However, surgical access to intraparenchymal tumors may vary according to the surgeon's experience and dexterity, technical possibilities, tumor location, and size. Arachnoid dissection, sulcal, and gyral dissection are generally used in direct transcortical approaches while reaching the tumoral tissue. Identification and preservation of anatomical land marks such as fiber tracts, arterial and venous vessels, and basal ganglia are the basis of the surgical strategy for preserving brain functions [(1–3)].

The developments in neuroanatomy, neurophysiology, and pathology, which started with the use of anesthesia and antisepsis in the early 1900s and continued with the use of radiography and new operating instruments, have developed modern neurosurgery (1). Today, a safe postoperative clinical outcome is provided by the analysis of preoperative imaging modalities, the evaluation of data obtained from intraoperative neurophysiological monitoring or imaging modalities (e.g., USG or MRI), and also postoperative ICP, EEG, and biochemical examinations (4, 5). Decision-making mechanisms for the approach to be used for brain tumors of different localizations and sizes are based on clinical guidelines, analyses, and statistics of previous cases.

Artificial intelligence (AI) has risen to prominence in the medical literature in recent years, and its usage is expanding beyond diagnosis to treatment on a daily basis. AI is the name given to machine systems, particularly computer systems, that can replicate human brain cognitive abilities such as learning, reasoning, and self-correction. In the simplest terms AI refers to systems or robots that resemble human intelligence to execute tasks and can improve themselves iteratively based on the data they collect. AI is working just like a simulation of the human brain intelligence and the goal of AI inspired by brain science is to develop systems that have features such as decision-making, lifelong learning, learning by association, long and short-term memory, recognition, classification of learned abilities, and interacting with the environment. In general, AI systems function by processing huge amounts of data, producing correlations and patterns, and using these outcomes to make predictions for future situations. In addition, AI has abilities such as creating and transferring knowledge and self-learning (6–8). Machine learning focuses on how to construct intelligent computer programs (or computational models) that automatically learn and understand the massive amounts of data and turn it into knowledge and action with experience (9).

There are two basic approaches based on the availability of labels in machine learning: supervised learning (labeled data) and unsupervised learning (unlabeled data). In supervised learning algorithms, the model tries to learn the relationship between the desired output and the input features (6). It can be used in daily practice for risk prediction (10) and reveals the effect of clinical prognosis by evaluating different factors (demographic or social changes) on treatment (6).

Unsupervised learning algorithms are mainly used to identify and investigate the unknown patternsin the input data. The model mines for rules, identify patterns, and summarizes meaningful findings (6, 7). On the other hand, unsupervised learning does not require prior knowledge of the output values and the data are unlabeled. When compared to supervised learning algorithms, these algorithms ensure more complex processing tasks. It can be used for diagnostics, patient selection, identifying symptom clusters associated with specific diseases, selecting optimal treatment strategies based on demographic or genetic features, pattern identification or recognition in radiological or photographic images, and other data in daily practice (6, 8).

Reinforcement learning is an interactive machine learning system (or framework) that detects how an AI agent takes action and interacts with the environment to make some sequence of decisions (6, 8, 9). It aims to achieve maximum reward from the rightful actions of the AI agent. AI agent takes action for reaching the goal by maximizing the total reward, and the RL model keeps continues to learn until finding the best solution. When the AI agent acts, it rewards the system for the correct output and punishes it for the incorrect output. RL can be used as a control algorithm to optimize the use of scarce resources in specific situations, such as the selection of patients to be discharged based on clinical logistics requirements and surgical aids or robots.

The classical neurosurgical techniques that reduce patient discomfort and the risk of neurological morbidity, provide shorter hospital stays, and the use of technological developments that support these techniques have been at the center of neurosurgery for decades (11–13). The cranial neurosurgical approaches and their modifications have been standardized for about the last 150 years (1, 11). This study aims to define a machine-learning algorithm to estimate optimal surgical pathways. By using a reinforcement learning approach to solve the path planning problem, the suggested method saves computational time by skipping unrelated cranial areas that were present in screenings while also boosting planned trajectory accuracy.

Q-learning is the most known and frequently used RL algorithm. In Q-learning, agent and environment are two important variables. The agent is an algorithm that takes actions based on the environment. The environment is the system in which the agent makes decisions and learns from its actions. The agent learns by interacting with its environment as a human would. The achievement of the agent's set goal is defined as the reward. Areas that should not go or touch are defined as penalty areas. Actions are defined as such activities performed by the agent. The reward is a measure of the success or failure of the agent. It follows by creating a reward table (14, 15). The agent begins searching for the target at random locations throughout the defined environment, analyzes its future steps, and records its successes and failures. This is the case until the agent discovers the first reward. As soon as the agent gets to a target location, it recalls its position before arriving at the target and records this value in the Q-Table where it has accumulated its own experiences (15–17). The agent develops policy, which is a decision-making strategy.

This article proposes a new heuristic-based surgical path planning algorithm for neurosurgery. The new heuristic estimates accurate optimal surgical paths avoiding critical structures in the brain. It computes the proper entry points on the scalp and then searches for different paths that reach the beginning location of the tumor and finds the optimal linear surgical paths. Then the extracted optimal linear paths from the new heuristic are used as an entry point or an environment [depending on the path width (dimension)] of the Q-learning algorithm for finding nonlinear access paths. Especially while finding nonlinear trajectories, usage of the new proposed heuristic's output can save computational time by skipping unrelated cranial areas that were present in screenings while also boosting planned trajectory accuracy. Moreover, the extracted nonlinear trajectories can improve clinical outcomes because they ensure minimally invasive approaches.

## Methods

The study involved a retrospective MRI analysis with no risk to the patients. This study was approved by the Institutional Clinical Non-Interventional Research Ethics Board (E-54022451-050.05.04-41353). These authors reviewed the cases together and reached a consensus in any disputed case. All procedures performed in studies involving human participants were in accordance with the ethical standards of the institutional and/or national research committee and with the 1964 Declaration of Helsinki and its later amendments or comparable ethical standards. For this type of study, formal consent was not required.

### Radiologic Imaging

#### MRI Technique and MR Tractography

MRI was performed with a 1.5-T system (Magnetom Avanto; Siemens, Erlangen, Germany). First, routine brain MRI protocol included T1-weighted (T1W; TR/TE = 460/14 ms) and T2-weighted (T2W; TR/TE = 2,500/80 ms) sequences in the axial and coronal planes, and fluid-attenuated inversion recovery (FLAIR) images (TR/TE = 8,000/90 ms) in the axial plane with 5-mm-thick sections. The DTI protocol consisted of a single-shot, spin-echo, echo-planar sequence with the fat suppression technique: TR/TE = 2,700/89 ms; matrix, 128 × 128; field of view, 230 mm; and slice thickness, 5 mm. DTI was acquired before the administration of contrast media and 30 diffusion-encoding directions were used at *b* = 1,000 s/mm^2^. After that, T1W 3D magnetization-prepared rapid gradient echo (TR/TE/TI = 12.5/5/450 ms) volumetric sequences with and without contrast medium (gadolinium-diethylenetriamine pentaacetic acid, 0.1 mmol/kg body weight, intravenously) was applied. The Syngo.*via* console (software version VB30A_HF06; Siemens) was used for the postprocessing of DTI data sets, after which the ADC and color-coded FA maps were reconstructed. Sending 30-way DTI images (30 diffusion-encoding directions) to Syngo.*via* console and performing tractography with MR Neuro 3D function. The DTI data sets and 3D MR images were analyzed using freeware for diffusion tensor analysis and fiber tracking (Syngo.*via* console). To depict the motor tracts, the seed area was placed on the cerebral peduncle where the corticospinal tract (CST) is known to run while observing the color-encoded fiber orientation map. Cortical target regions were carefully placed in the suspected primary motor area. We used the two-regions-of-interest method (i.e., seed and target regions) to demonstrate on—well the descending fibers from the primary motor area to the cerebral peduncle.

In MR tractography, the blue coding shows the corticospinal tract with a top-down (craniocaudal) course, the red coding for the transverse course in the corpus callosum and subcortical areas, and the green coding for the anteroposterior front-occipital tracts.

### Algorithms

**Figure 1:**
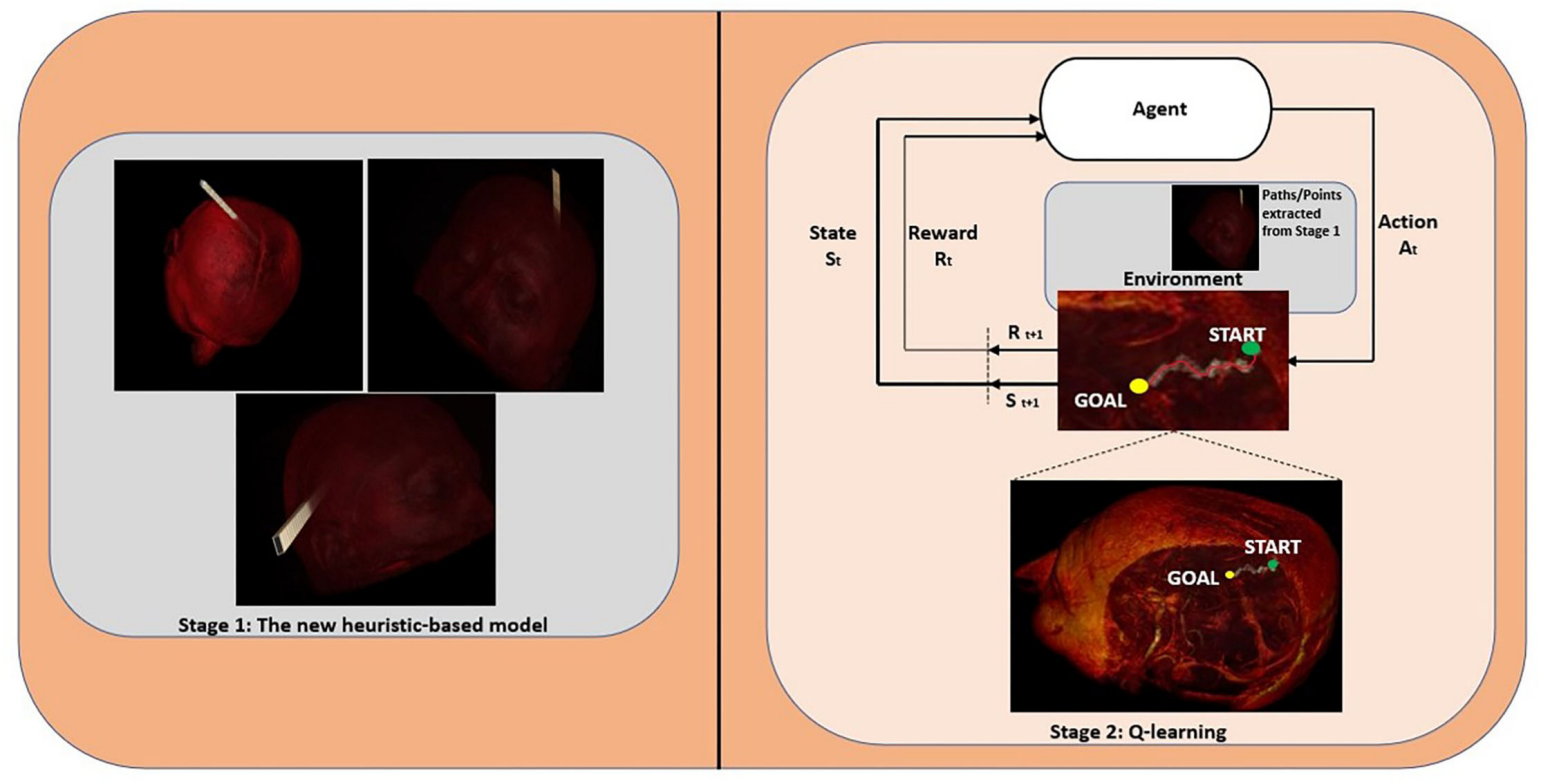
The proposed system architecture for finding linear and nonlinear access paths for neurosurgery.

By using all the new heuristic and Q-learning algorithms together, we extract not only linear but also nonlinear access paths. Our model works in two stages, in the first stage the new heuristic is used to find linear paths and in the second stage the Q-learning algorithm is used to find nonlinear paths. We also handle different path dimensions (i.e., each cell contains *nxn* points) such as 16 × 16, 32 × 32, 40 × 40, and 64 × 64 to prove the accuracy of our method. Especially for 16 × 16 and 32 × 32 dimensions, the pathways are so narrow, whence the entry points of these paths are taken as reference entry points for the Q-learning algorithm. For larger dimensions, the extracted paths are used as an environment of the Q-learning algorithm, and then the AI agent takes action and finds the best possible nonlinear surgical access paths. Figure 1 illustrates the proposed system architecture for finding linear and nonlinear access paths for neurosurgery.

In the first stage (Stage 1), the proposed heuristic-based algorithm, first, the three dimensions (*x, y, z*) of given MRI images in DICOM format are extracted. The first two dimensions denote the matrix rows and matrix columns in pixels, respectively and the third dimension denotes the number of axial T1-weighted MR images of the patient with brain tumors. After that, all the surfaces (top, bottom, right-side, left-side, front, and back) of the axial T1-weighted MR images are divided into cells (e.g., each cell contains 16 × 16 points), and then it is calculated how many cells can take place in these DICOM files. For each dimension of the given MR images, different processes are utilized to calculate all points over each cell, i.e., the *x*-axis, *y*-axis, and *z*-axis do not change for the top and bottom surfaces, the left-side, and right-side surfaces, and front and back surfaces, respectively. The number of total cells gives all the possible starting entry points. Later, the proposed algorithm searches for all paths that reach the beginning location of the tumor (the coordinate points for the tumor and eloquent areas are labeled with Labelme [(18); https://github.com/wkentaro/labelme] which is an image annotation tool) (Algorithm 1 in Supplementary Material), and then the coordinate points in each path are extracted (Algorithm 3 in Supplementary Material). Because of the huge search space and the computation time cost, while calculating the optimum paths the number of all the possible starting entry points has been reduced in four stages. In every stage, the coordinate points over the calculated paths are compared with the labeled critical structures one by one, if these points intersect with these structures the penalty score of the related path is increased. At the end of the comparison, all penalty scores are sorted in ascending order. Finally, at the end of the fourth stage, the top 20 (this number of paths is optional and can be changed) paths are extracted (Algorithm 5 in Supplementary Material).

The new heuristic-based surgical path planning algorithm finds the best optimal n-paths which include all the path points (coordinates), then these paths are used in the second stage (Stage 2–Q-learning algorithm) for finding nonlinear paths. The Q-learning algorithm seeks to find the best action in the given current state. The Q-table is utilized to select the best action based on the *q*-value, and after each episode, it is updated with the new *q*-values. While updating *q*-values, they are adjusted based on the difference between the new *q*-value and old *q*-value by using “learning rate” and “discount factor” parameters. The learning rate is defined as the weight of how much we consider the new value, and the gamma is a discount factor and balances the immediate and future reward. With the “np.max” function, the maximum of the future reward is taken, and this value is applied to the reward for the current state (Eq. 1).

**NewQ[state, action] = Q[state, action] + learning rate** ^*^ **(reward + gamma** ^*^ **np.max(Q[new_state,:]) — Q[state, action])** (Eq.1)

The agent interacts with the environment (is extracted in Algorithm 6 in Supplementary Material) in two ways exploiting (selecting action with the highest value by referencing the Q-table) and exploring (random action selection). Before starting the Q-learning algorithm, all the points (coordinates) on the paths are classified by using the corresponding labeled structures. Thus, the points of all structures belonging to the same class (label value) in different layers are gathered under an array. All the points are considered as a node, and then the reward and penalty scores are assigned to nodes in each class by considering the penalty score of the critical structures, and “1” penalty score is assigned to all other nodes where the agent can interact in. Each node has a neighboring node. If a node is not on the edge or corner, it has eight neighboring nodes (right, left, up, down, bottom-right, bottom-left, top-right, and top-left) over the same layer. The same node has 18 different neighboring nodes over the one upper layer (9 neighboring nodes) and one lower layer (9 neighboring nodes) (**Figure 5**). The penalty scores of all these 27 different nodes are assigned by considering the penalty score of each class. Then Q-learning algorithm is executed over these nodes to find the best possible nonlinear surgical access paths (Algorithm 7 in Supplementary Material).

### Experimental Results

To find the best possible linear and nonlinear surgical paths, the proposed new heuristic and Q-learning algorithms were executed, respectively. As a case study, we used (512 × 512 × 144) axial T1-weighted MRI images of one patient with a brain tumor in DICOM format, then 16 × 16, 32 × 32, 40 × 40, and 64 × 64 path dimensions were evaluated for the new heuristic algorithm. These dimensions correspond to the cell parameter (*n* is equal to 16, 32, 40, and 64, respectively in each case) given in Algorithm 5 in Supplementary Material. For each case, we extracted 20 optimal linear paths, and the extracted linear paths for 16 × 16 and 32 × 32 dimensions were so narrow for executing the Q-learning algorithm. So, we used the entry points of these paths as reference starting (entry) points of the second stage. For the 40 × 40 and 64 × 64 dimensions, each 20 extracted linear path composed the environment of Stage 2. While finding linear paths, the heuristic checked millions of coordinate points. All steps given in Algorithm 5 in Supplementary Material were applied one by one to 16, 32, 40, and 64 cell dimensions. We picked 80, 40, and 20 optimal paths for the second, third, and fourth path index sequences, respectively. The findings in the intermediate steps are given in **Table 2**. The first column in the table gives information about the intermediate steps in Algorithm 5 in Supplementary Material: the number of all possible paths, the number of checked points in the sequence, and the chosen parameters to indicate the number of optimal paths. The second column in the table gives more details for each cell dimension (**Table 2**).

While the total of 40 linear paths extracted from the 16 × 16 and 32 × 32 dimensions was used as reference starting points in Stage 2, the total of 40 linear paths extracted from the 40 × 40 and 64 × 64 dimensions was also used as an environment. Then the extracted nonlinear paths were observed by neurosurgeons. The best path was obtained from the 16 × 16 dimension and are given in **Figure 4**.

## Discussion

In this study, we proposed new system architecture which includes two stages to find linear and nonlinear access paths for neurosurgery. In the first stage of the model, linear paths were found, then these paths/or entry points were utilized for finding nonlinear paths in the second stage. We proposed a new heuristic-based surgical path planning algorithm for finding linear paths. Moreover, the Q-learning algorithm was used to find the nonlinear access paths by using the extracted linear paths from the new heuristic. We performed a Q-learning algorithm over cranial MRI scans to learn which steps ensure reaching the beginning location of the tumor with maximum rewards. Touching the critical structures is assigned as a penalty while reaching the tumor tissue is defined as a reward. As an environment, the entire head is taken into account, so the possibility of nontraditional surgical methods was not ignored (different access routes that may be suitable for open face or endoscopic procedures). Some neural fiber routes, arteries, veins, and dural sinuses have been identified as structures that should be avoided (Table 2). The algorithm aims to reach the tumor while avoiding these critical locations (evaluated as penalty scores), and uses Q-Table helps to find the best action for each state in the brain. The state that expresses the current status (position) of the AI agent in the environment is the *x, y, z* coordinated location in cranial MRI (14–17).

**Table 1:** Some major surgical landmarks and their functions for transcortical approaches.

**Approach**			
**Frontal**	Gyrus Rectus, Distal AntComA	
	Caudate nucleus	Bottom up attention (goal directed), memory, learning, sleep, emotion, language
	Fornix	Memory
	Inferior Frontal Gyrus (pars opercularisandriangularis)	Langage (If), theory of mind (bilat), visuospatial cognition (rt)
	Anterior perforate substance Optic tract Precentral gyrus Broadman 44	Corticospinal tract vascular supply
**Temporal**	Crus cerebri, pca, uncus	
	Lateral sulcus	
	Optic radiation Hippocampus	Optic pathway Memory pathway
	Visual word form area	Identifying words
	Arcuate fasciculus	Language (It), visuospatial cognition (rt)
	IFOF cuneus	Language (It), visuospatial (rt)
**Parietal superior**	Superior anastomotic vein (trolard)	
	Postcentral gyrus	Sensitive patnway
	Parietal operculum	Sensitive pathway
	Heschl's gyrus	Connection speech
	Superior longitudinal fasciculus Ill	Language (It), visuospatial (rt)
	Arcuate fasciculus Language	Language (It), visuospatial (rt)
**Insula**	Periinsular sulcus	
	Lenticular nucleus	
	Arcuate fasciculus (lat to claustrum)	Language (If), visuospatial (rt)
	IFOF (btw claustrum and putamen)	Language (If), visuospatial (rt)
**Veins**	Vein of Labbé (inferior anastomotic vein)	Temporoparietal drainage
	Basal vein of Rosenthal	
	Superficial sylvian vein	
	Superior sagittal sinus and another main sinuses	main venous drainage
**Arteries**	ICA and main branches	
	Basiler arter and main branches	

**Table 2:** Gives the details in the intermediate steps of the proposed heuristic for the case study.

	**Cell Dimension**			
	**16 × 16**	**32 × 32**	**40 × 40**	**64 × 64**
The number of all possible paths	745,984	675,840	641,920	544,768
The *n-*optimal paths in the “SECOND_PATHS_INDEX” sequence	80	80	80	80
The number of checked points in “SECOND_PATHS_INDEX” sequence	46,727,360	83,222,240	112,560,000	76,212,160
The *m-*optimal paths in the “THIRD_PATHS_INDEX” sequence	40	40	40	40
The number of checked points in “THIRD _PATHS_INDEX” sequence	58,339,960	146,084,080	137,648,480	85,084,960
The *l-*optimal paths in the “FOURTH_PATHS_INDEX” sequence	20	20	20	20

*Number of MRI slice*.

**Figure 2:**
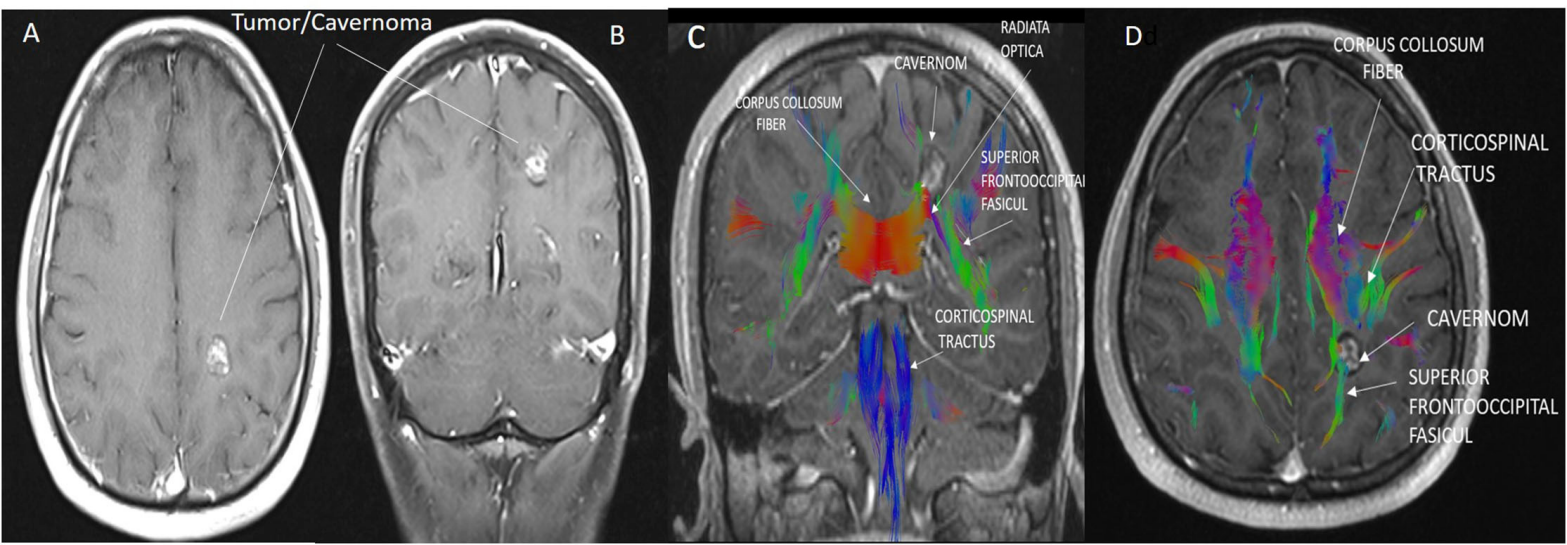
**(A,B)** Cavernoma appearance on axial **(A)** and coronal **(B)** contrast-enhanced T1 cranial MRI images. **(C,D)** The anatomical relationship of the corticospinal tract, superior fronto occipital fasciculus, and corpus callosum transverse fibers with the cavernoma is shown in sagittal and axial MRI tractography images. Due to the mass effect of the cavernoma, displacement of the superior fronto occipital fasciculus was observed.

**Figure 3:**
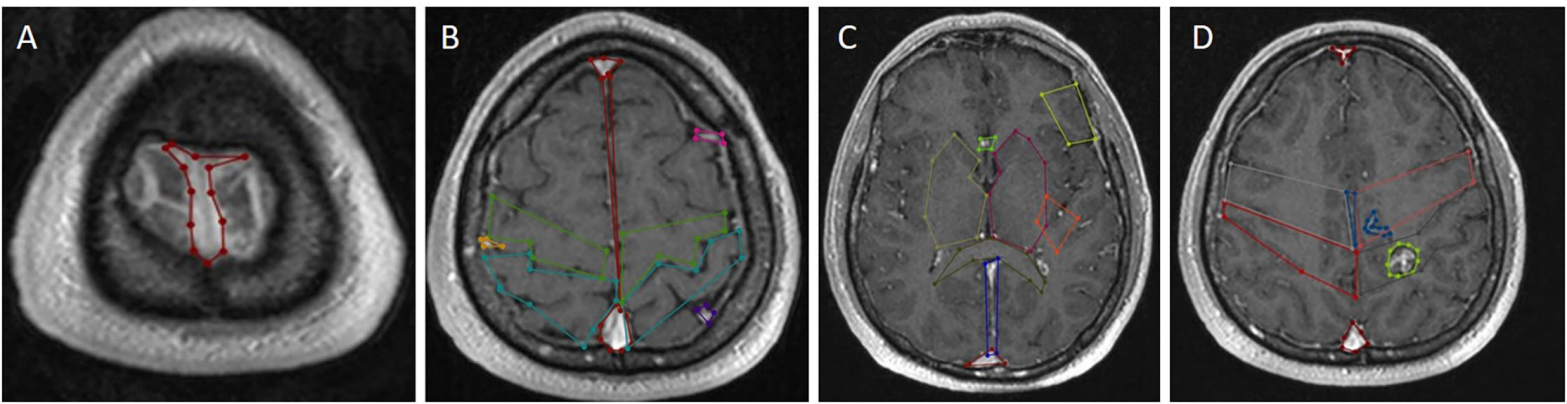
Labeling using contrast-enhanced T1 axial image of cranial MRI. **(A)** Superior sagittal sinus marked in red at the vertex's midline. **(B)** Superior sagittal sinus marked with red in the midline in the supraventricular area, precentral gyrus marked with green, postcentral gyrus marked with turquoise, superficial cortical veins marked with pink on the left and dark yellow on the right adjacent to the bilateral frontal lobes. **(C)** Right basal ganglia and thalamus marked with yellow in the right cerebral hemisphere at the ventricular level; left basal ganglia and thalamus marked with light red in the left cerebral hemisphere at the ventricular level, Broca's area in the left frontal lobe with light yellow, Wernicke's area posterior to Sylvian fissure marked with orange; The anterior cerebral arteries are marked in light green anteriorly in the midline, the corpus callosum splenium in green and the sinus rectus in blue in the midline posteriorly. **(D)** Right postcentral gyrus marked red, cavernom/tumor marked yellow-green, pericallosal artery marked blue on the midline and posterior inferior frontal artery marked blue.

The borders of the preoperative brain tumor lesion (reward) were determined as the contrast-enhancing area in T1 contrast MRI imaging (19). Tracts with diffusion tensor imaging (DTI), arterial anatomy with contrast-enhanced MRI angiography (CE-MRA), and those with tumor tissue (20) superficial cortical vessels and dural sinuses are used in surgical planning with MRI venography (21) (Figure 2). DICOM images in the respective sequences were imported into the labeling program (labelme 4.6.0, https://github.com/wkentaro/labelme). Functional anatomical areas were marked and labeled by the radiology and neurosurgeon specialist (Figure 3). The pixel and voxel values of the anatomical point and the anatomical structure of the point were listed by labeling (22, 23). This gave us the advantage of trading in a cubic system with matrixes.

**Figure 4:**
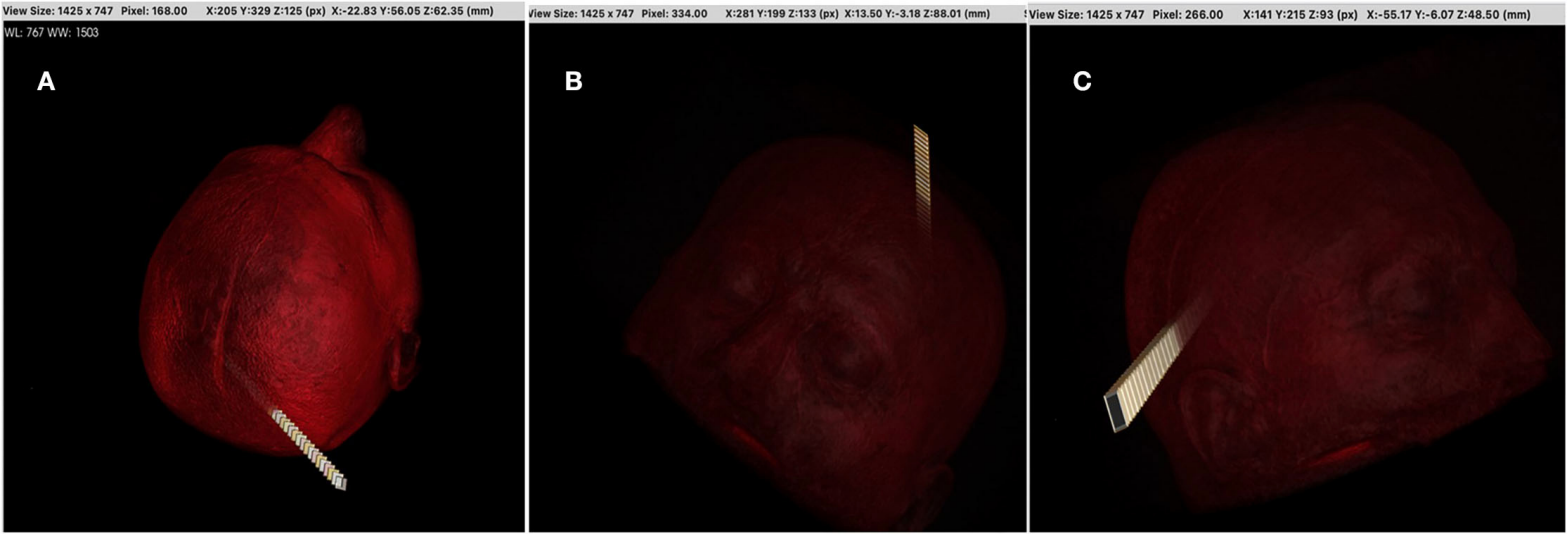
The research algorithm was created for time efficiency compared with the time-consuming RL algorithm. The goal is to find the most ideal cranial entry points. Machine learning was not used in this method. Cranial entry points were scored using the equivalent areas and tumor location in Table 1 and compared with each other. With this algorithm, it was possible to sort by five most ideal entry points, 10 entry points, or worst entry points. In addition, this algorithm provided a linear access path to tumor tissue in the shape of a rectangular prism or cylinder. The entrance area in the images was determined as 1.5 cm^2^. The algorithm has been adjusted to allow this area to be increased or decreased. This algorithm can be useful in tubular operative systems or rigid endoscopic systems. In this study, we took these points (the most ideal 4,900 points) as the starting points of RL. Image **(A,B)** are the ideal best rated and image **(C)** the worst-rated sample entry points.

**Figure 5:**
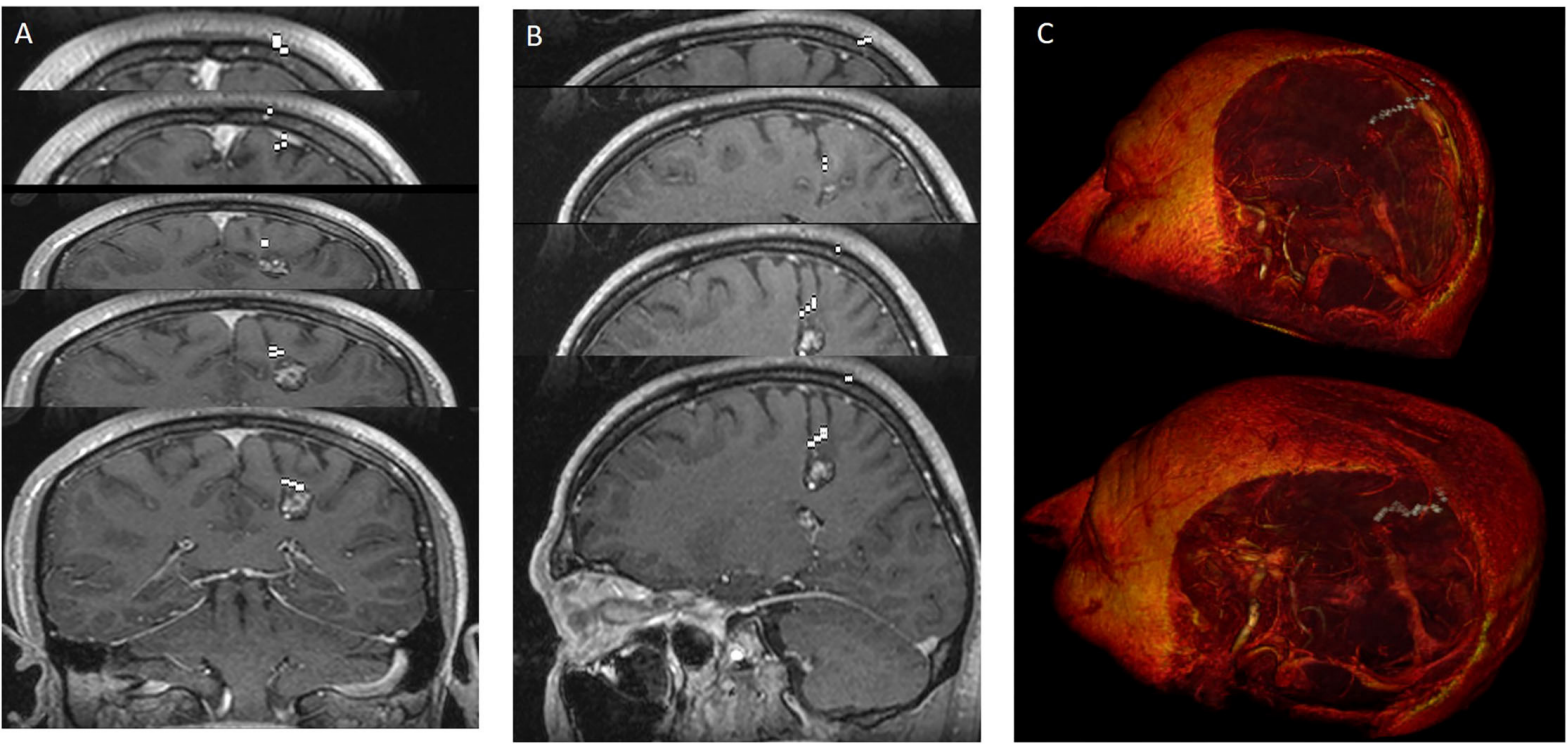
The most ideal cortico-tumoral approach is recommended by RL. Images were added one after another to show the nonlinear pathway. RL extracted the most optimal pathway by performing a random-onset point analysis of the entire intracranial area. Demonstration of the approach reaching the tumor from the base of the postcentral sulcus**. (A)** howing the pathway in coronal sections. **(B)** Showing the pathway in sagittal sections. **(C)** Showing the 3-dimensional pathway with image processing.

**Figure 6:**
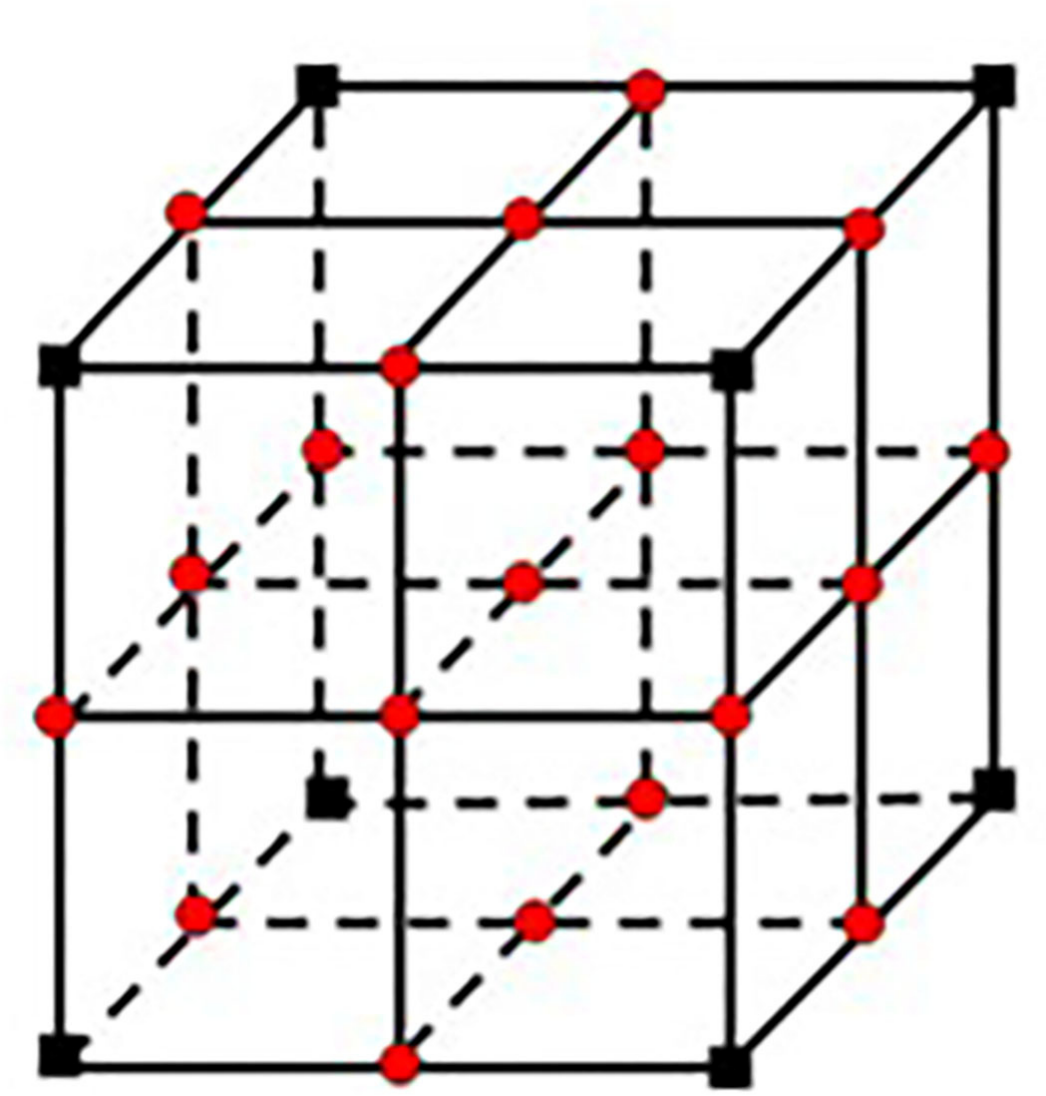
The figure illustrates the proposed system architecture for finding linear and nonlinear access paths for brain surgery.

We used (512 × 512 × 144) axial T1-weighted MRI images of one patient with a brain tumor in DICOM format as a case study. We utilized 16 × 16, 32 × 32, 40 × 40, and 64 × 64 path dimensions to evaluate the success of the proposed system architecture. In the first stage, 20 optimal linear paths were extracted for each dimension by using the new heuristic-based algorithm. The optimal nonlinear path was extracted by using the starting points found in the 16 × 16 path dimension. For the 16 × 16 path dimension, the proposed heuristic found 745,984 possible entry points. The areas in Table 2 and the target tumor tissue were accepted as reference points. This algorithm gave input fields of desired diameter and size. These 745,984 linear paths were compared according to the reward and penalty points. By using the intermediate steps, the 20-optimal linear paths were chosen. Then the starting points of these paths were used as reference entry points of the Q-learning algorithm (Figure 4) in the second stage. Then, a matrix size of 78,030 × 78,030 was created and worked on 50 × 25 × 78,030 points for Q-learning. Extracranial areas were excluded. It was enough to find 500,000 epoch paths in a 16 × 16 × 35 environment, and it almost took 70 min. The Q-learning algorithm returns as the best way “node” (Figure 5). Then, the positions of these node values in the matrix were found and the *x, y*, and *z* coordinate values were reached. Thus, the coordinates representing the best path were extracted from the DICOM images. The most ideal transcortical tumoral pathway was revealed in Figure 6.

On the other hand, all the paths have been shown in DICOM format, thus the usage of MR images in DICOM format has provided data protection while also allowing us to use it in postprocessing systems (OsiriX MD v12.5.0–SNRTech Workstation). One of the achievements of this study is that all the extracted linear and nonlinear paths in DICOM format can be seen in any neuronavigation software. The neuronavigation device utilized in the operating room has been evaluated for availability, but the proposed system is not yet used on a patient during surgery (neuronavigation system). Following the requisite assessment and approval, future work would be used as an intraoperative guide.

The number of studies on artificial intelligence and neurosurgery has risen considerably in recent years (24, 25). In this article, the proposed artificial intelligence-based system architecture was utilized to find the most optimal surgical paths for preoperative patient images that were labeled on the 3-dimensional coordinate plane with cranial MRI voxel values. The results (linear and nonlinear paths) obtained with this basic system architecture (framework) can be used with intraoperative neuronavigation as well as road maps in tubular, endoscopic, robotic, and augmented reality.

The mathematical equation of any case provides the advantage of measure and control tools to use in. Many current technical types of equipment and treatment algorithms have evolved through the mathematical equations defined in the past centuries. Introducing Preoperative neuroanatomical points and functions as data to artificial intelligence will highlight patient-specific surgical approaches. The near future will be based on artificial intelligence, which will employ an excessive amount of data, and the relationship between these data will allow surgery to be performed with greater accuracy.

Perioperative monitoring and use of cranial anatomy and functions contribute to a positive outcome. In future work, new methods based on artificial intelligence would be used to analyze data for each stage of neurosurgical interventions, and these initiatives would be maximized. Simultaneously, that will allow patients to benefit from the experience of an unlimited number of clinics and surgeons in personal treatment planning.

### Limitation of Study

The first limitation is the manual segmentation of anatomical points. This took a lot of time for both the surgeon and the radiologist. Points were marked in 144 axial images. The second limitation was that we could not create fusion MRI images. We aimed to make a single fusion MRI sequence of T1 contrast axial, DTI, TOF, and venography sequences and use it. However, we could not use the fusion images we obtained as DICOM data. Fusion sequence MRI would enable us to use anatomical accuracy at a single point level. This caused the third limitation of the study, the necessity of specifying four points in the labeling. It made the processing points coarser and larger. Our fourth limitation is the total processing time. We think that when the first three limitations are resolved and the computer processor speeds increase, the processing time will be shortened.

To reduce the computational cost, we did not handle all of the linear paths extracted from the heuristic, and we eliminated most of these paths. In our experiments, we have used a 10-core CPU with eight performance cores and two efficiency cores, 16-core GPU, 16-core Neural Engine, 200 GB/s memory bandwidth, and OS X. We think that by using the more powerful computers all these linear paths can be used and then the found all possible linear paths can be used reference points/environments based on the cell dimension for Stage 2. We also gave a case study to observe the achievement of our system. We used (512 × 512 × 144) axial T1-weighted MRI images of one patient with a brain tumor in DICOM format, then 16 × 16, 32 × 32, 40 × 40, and 64 × 64 path dimensions were evaluated for the new system. The best optimal nonlinear surgical path was found from the 16 × 16 dimension. There are some studies (Liedlgruber et al.) (26) in the literature that use automatic segmentation, but vascular structures are not taken into consideration in these segmentations. Tomasi et al. (27) combined the cerebral cortex anatomy and vascular structures in their studies. In future work, we plan to use automatic segmentation by taking into consideration vascular structures in the brain.

## Results

In this article, a new system architecture based on a two-stage is proposed to find linear and nonlinear access paths for neurosurgery. In the first stage, the proposed new heuristic estimates accurate optimal surgical paths avoiding critical structures in the brain. It computes the proper entry points on the scalp and then searches for different paths that reach the beginning location of the tumor and finds the optimal linear surgical paths. In Stage 2, the extracted optimal linear paths from the new heuristic are used as an entry point or an environment for the Q-learning algorithm to find nonlinear optimal paths. Artificial intelligence has the potential to reduce medical errors while also reducing healthcare costs. It will be based on artificial intelligence in the near future, where big data will be used, the relationship between these algorithms and neuroanatomical functions are determined more precisely and neurosurgery can be performed with them.

## Data Availability Statement

The original contributions presented in the study are included in the article/Supplementary Materials, further inquiries can be directed to the corresponding author.

## Ethics Statement

This study was approved by the Institutional Cinical Non-Interventional Research Ethics Board (E-54022451-050.05.04-41353). All procedures performed in studies involving human participants were in accordance with the ethical standards of the institutional and/or national research committee and with the 1964 Declaration of Helsinki and its later amendments or comparable ethical standards. For this type of study, formal consent was not required.

## Author Contributions

TD and ND conceived and designed the analysis. TD, MP, and AM wrote the article. SK, ID, AG, TD, and UY performed the analysis. TD, MD, MK, RT, and ND collected the data. AE, UY, MK, RT, MD, and ND created the algorithms. All authors contributed to the article and approved the submitted version.

**Algorithm 1**. Algorithm 1 gives the pseudocode for finding the number of all paths that reach the beginning location of the tumor (defined as goalPoint). The given MR images handle as a square prism, then all possible paths have been calculated by using the cell dimensions for six surfaces of the prism.

**Algorithms 2 and 3**. Algorithm 3 gives the pseudocode for finding all the coordinate points inside each path. The input of Algorithm 3 is the output sequence of Algorithm 1. The algorithm also uses Algorithm 2 which finds the area of the selected part.

**Algorithms 4 and 5**. Algorithm 5 defines the steps of our new heuristic and gives the pseudocode for finding the optimal n-paths. It also uses the given other algorithms. The output of the heuristic is the n-paths with minimum penalty scores which have been calculated by using Algorithm 4.

**Algorithms 6 and 7**. Algorithm 6 gives the steps of how optimal linear paths (extracted from Algorithm 5) are converted to an environment. Algorithm 7 (simply Q-learning algorithm) gives the steps of finding the best optimal paths by interacting with the found environment (Algorithm 6).

## Conflict of Interest

The authors declare that the research was conducted in the absence of any commercial or financial relationships that could be construed as a potential conflict of interest.

## Publisher's Note

All claims expressed in this article are solely those of the authors and do not necessarily represent those of their affiliated organizations, or those of the publisher, the editors and the reviewers. Any product that may be evaluated in this article, or claim that may be made by its manufacturer, is not guaranteed or endorsed by the publisher.
