# Supplementary material for: Machine Learning-Based Surgical Planning for Neurosurgery: Artificial Intelligent Approaches to the Cranium
Source: Front Surg. 2022 Apr 29;9:863633. doi: 10.3389/fsurg.2022.863633 (PMC9099011; doi:10.3389/fsurg.2022.863633)
Supplement: Supplementary file 1 [file Data_Sheet_1.docx]

| **Algorithm 1** Find the Number of All Paths |
| --- |
| **Input:** The (x, y, z) dimensions of given MR images and the dimension of cell and the beginning location of the tumor;  **Output:** The number of All Paths **FindAllPaths**(coords, cell, goalPoint); |
| 1: x, y, z$\leftarrow$coords; |
| 2: **for** j in [0, x - cell) finding the paths on the front surface |
| 3: **for** k in [0, y - cell) |
| 4: front$\leftarrow$[ frontpathPoints, goalPoint ] ; allResult $\leftarrow$front the paths on the front surface |
| 5: back$\leftarrow$ [backpathPoints, goalPoint ]; allResult $\leftarrow$back  the paths on the back surface |
| 6: **end for** |
| 7: **end for** |
| 8: **for** j in [0, y - cell) finding the paths on the top surface |
| 9: **for** k in [0, z - cell) |
| 10: top$\leftarrow$ [toppathPoints, goalPoint ]; allResult $\leftarrow$top  the paths on the top surface |
| 11: bottom$\leftarrow$[bottompathPoints, goalPoint]; allResult$\leftarrow$bottom the paths on the bottom surface |
| 12: **end for** |
| 13: **end for** |
| 14: **for** j in [0, x - cell) finding the paths on the left-side surface |
| 15: **for** k in [0, z - cell) |
| 16: left $\leftarrow$ [leftpathPoints, goalPoint ]; allResult $\leftarrow$left  the paths on the left-side surface |
| 17: right$\leftarrow$[rightpathPoints, goalPoint ]; allResult$\leftarrow$right  the paths on the right-side surface |
| 18: **end for** |
| 19: **end for** |
| 20: return shape.allResult the number of all paths |
