# Supplementary material for: Machine Learning-Based Surgical Planning for Neurosurgery: Artificial Intelligent Approaches to the Cranium
Source: Front Surg. 2022 Apr 29;9:863633. doi: 10.3389/fsurg.2022.863633 (PMC9099011; doi:10.3389/fsurg.2022.863633)
Supplement: Supplementary file 2 [file Data_Sheet_2.docx]

| **Algorithm 2** Find the area of the selected area |
| --- |
| **Input:** the selected area;  **Output:** the area of the selected area **FindArea**(selectedArea); |
| 1: sortedRow $\leftarrow$ sorted( selectedArea, key=lambda x: x[0], reverse=False ) |
| 2: x $\leftarrow$ sortedRow[0][0] |
| 3: y $\leftarrow$ sortedRow[-1][0] |
| 4: sortedCol $\leftarrow$ sorted(selectedArea, key=lambda x: x[1], reverse=False) |
| 5: a $\leftarrow$ sortedCol[0][1] |
| 6: b $\leftarrow$ sortedCol[-1][1] |
| 7: tempAllPoints $\leftarrow$ [ ] |
| 8: **for** i in ( x, y + 1 ) |
| 9: **for** j in ( a, b + 1 ) |
| 10: tempAllPoints $\leftarrow$ [i, j] |
| 11: **end for** |
| 12: **end for** |
| 13: **return** tempAllPoints |

| **Algorithm 3** Find the Coordinate Points in Each Path |
| --- |
| **Input:** the output sequence of the **FindAllPaths** function;  **Output:** the coordinate points in each path **FindPointsInPaths**(paths); |
| 1: cent $\leftarrow$( sum ([p[0] for p in paths]) / len(paths), sum( [p[1] for p in paths] ) / len(paths) ) |
| 2: paths $\leftarrow$ sort(key=lambda p: math.atan2(p[1] - cent[1], p[0] - cent[0]))  the distance of each point to the middle point of the polygon (ascending order) |
| 3: allPoints $\leftarrow$ **FindArea**(paths)  Algorithm 2 |
| 4: result $\leftarrow$[ ] |
| 5: polygon = **Polygon**(paths)  **Polygon** method creates a square area with the given points |
| 6: **for** i in allPoints |
| 7: point = **Point**( i[0] , i[1] )  **Point** method defines a point in space with two given coordinates |
| 8: a = **polygon.contains**(point)  **polygon.contains** method checks whether the given point is inside the polygon and returns Boolean value |
| 9: b = **polygon.touches**(point)  **polygon.touches** method checks whether the given point is on the polygon and returns Boolean value |
| 10: **if** a **or** b |
| 11: result $\leftarrow$ i |
| 12: **end if** |
| 13: **end for** |
| 14: **return** result |
