# Supplementary material for: Machine Learning-Based Surgical Planning for Neurosurgery: Artificial Intelligent Approaches to the Cranium
Source: Front Surg. 2022 Apr 29;9:863633. doi: 10.3389/fsurg.2022.863633 (PMC9099011; doi:10.3389/fsurg.2022.863633)
Supplement: Supplementary file 3 [file Data_Sheet_3.docx]

| **Algorithm 4** Find Cost Sequence |
| --- |
| **Input:** The path sequence and the coordinate points sequence;  **Output:** Find Cost Sequence **FindCostSeq**(paths, points); |
| 1: *counter*$\leftarrow$ 0; |
| 2: *inner_points*$\leftarrow$ 0; |
| 3: *penalty_score*$\leftarrow$ 0; |
| 4: *labeled_data*$\leftarrow$the coordinates of the labeled data; Extracted from the CSV file of Labelme tool |
| 5:  **for** each *path* within the *paths* sequence **do** |
| 6: **if** *points* are in *labeled_data* **then** |
| 7: *inner_points* $=$ *inner_points* $+1;$ |
| 8: **if** the label of *points* in the *paths* is equal to the “Cavernoma” label **then** |
| 9: *counter* $=$ *counter*$+1;$ |
| 10: **end if** |
| 11: **end if** |
| 12: *penalty_score* $=$( *inner_points - counter*) $\times$ 10; |
| 13: *cost*$\leftarrow$ *penalty_score* of each *path*; |
| 14: counter$\leftarrow$ 0; *inner_points*$\leftarrow$ 0; *penalty_score*$\leftarrow$ 0; |
| 15: **end for** |
| 16: **return** *cost*; |
| **Algorithm 5** Search Optimal n-Paths |
| **Input:** T1-weighted MR images in DICOM format of a patient with brain tumors;  **Output:** Reduced Optimal Paths **FindOptPaths**(); |
| 1: *coords*$\leftarrow$x,y,z; Extract the three dimensions (x, y, z) of given MR images; |
| 2: *cell*$\leftarrow$*n*; |
| 3: *counter*$\leftarrow$ 0; |
| 4: *inner_points*$\leftarrow$ 0; |
| 5: *penalty_score*$\leftarrow$ 0; |
| 6: *goalpoint*$\leftarrow$ the beginning location of the tumor (x,y,z) |
| 7: *paths*$\leftarrow$ **FindAllPaths**(coords, cell, goalpoint); Algorithm 1 |
| 8: *points*$\leftarrow$**FindPointsInPaths**(paths); Algorithm 3 |
| 9: *second_points*$\leftarrow$pick the 10% of points in the *paths* in *points* sequence; (*10% is optional and can be changed*) |
| 10: *cost*$\leftarrow$ **FindCostSeq**(*paths*, *first*_*points*); Algorithm 4 |
| 11: *second_paths_index*$\leftarrow$all penalty scores in the *cost* sequence are sorted in ascending order and then write the index values of the number of 20% of all paths with the lowest penalty scores; (*20% is optional and can be changed*) |
| 12: *second_points*$\leftarrow$pick the 50% of points in the *paths* in *second_paths_index* sequence; (*50% is optional and can be changed*) |
| 13: *cost*$\leftarrow$ **FindCostSeq**(*second_paths_index*, *second_points*); |
| 14: *third_paths_index*$\leftarrow$all penalty scores in the *cost* sequence are sorted in ascending order and then write the index values of the number of 10% of all paths with the lowest penalty scores; (*10% is optional and can be changed*) |
| 15: *third_points*$\leftarrow$pick the all of the points in the *paths* in *third_paths_index* sequence; |
| 16: *cost*$\leftarrow$ **FindCostSeq**(*third_paths_index*, *third_points*); |
| 17: *fourth_paths_index*$\leftarrow$all penalty scores in the *cost* sequence are sorted in ascending order and then the top *10* (*this number of paths is optional and can be changed*) paths are extracted; |
| 18: *seq*$\leftarrow$*fourth_paths_index* |
| 19: **return** *seq*; |
