# Supplementary material for: Machine Learning-Based Surgical Planning for Neurosurgery: Artificial Intelligent Approaches to the Cranium
Source: Front Surg. 2022 Apr 29;9:863633. doi: 10.3389/fsurg.2022.863633 (PMC9099011; doi:10.3389/fsurg.2022.863633)
Supplement: Supplementary file 4 [file Data_Sheet_4.docx]

| **Algorithm 6**  Creating a matrix with neighborhoods and points for Q-Learning Algorithm |
| --- |
| **Input:** T1-weighted MR images in DICOM format of a patient with brain tumors and Reduced Optimal Paths;  **Output:** Creating a R matrix **CreateRMatrix(**labeled_data, points, detail_area**);** |
| 1: *labeled_data*__the coordinates of the labeled data; Extracted from the CSV file of Labelme tool |
| 2: *labeled_node* __labeled_data turned into unique nodes |
| 3: *cavernoma_array*__Nodes labeled cavernoma in each layer were added to the cavernoma array |
| 4: *block_array*__ Nodes with other tags in each layer were added to the blocks array |
| 5: *detail_area*__ the coordinates of paths from **FindOptPaths()** Algorithm 5 |
| 6: *R_memmap_matrices* __ A Matrix holding the score and neighborhoods points |
| 7: *points*__**FindPointsInPaths**(detail_area); Algorithm 3 |
| 8: *nodes*__ points turned into unique nodes |
| 9: *edge_list*__ List of all neighborhoods for each *nodes* in 3D environment |
| 10: goal_score= 0.1, block_score=10, normal_score=1 |
| 11: *R_memmap_matrices* .zeros |
| 12: **for** edge in (*edge_list*) |
| 13: **if** the *labeled_node* of *nodes* in the *edge_list* is in *cavernoma_array* **then** |
| 14: R_memmap_matrices.value =goal_score |
| 15: **end if** |
| 16: **if** the *labeled_node* of *nodes* in the *edge_list* is in *block_array* **then** |
| 17: R_memmap_matrices.value =block_score |
| 18: **end if** |
| 19: **if** there is *edge* are not in any list |
| 20: R_memmap_matrices.value =normal_score |
| 21**: end if** |
| 22: **end for** |
| 23: **return** R_memmap_matrices.value; |

| **Algorithm 7** Q-Learning Algorithm for Finding Optimal Paths |
| --- |
| **Input:** R_memmap_matrices from **CreateRMatrix**  **Output:** Finding path with best Q-value scores **FindPathsQL(**R_memmap_matrices**);** |
| 1: A= State has a R_memmap_matrices.values>0 __ Available actions, a= current action |
| 2: S= current state , S’= next state |
| 3: Set parameters: learning_rate=0.1 , discount_factor = 0.8, epsilon=0.9, epsilon_decay=0.995, epsilon_min: 0.01 , epoch_number= 700000 |
| 4: R= R_memmap_matrices.values__ Reward |
| 5: Initialize Q(S,A) __ Create Q table with all states, set 0 all score |
| 6: **for** i in range(epoch_numbers): _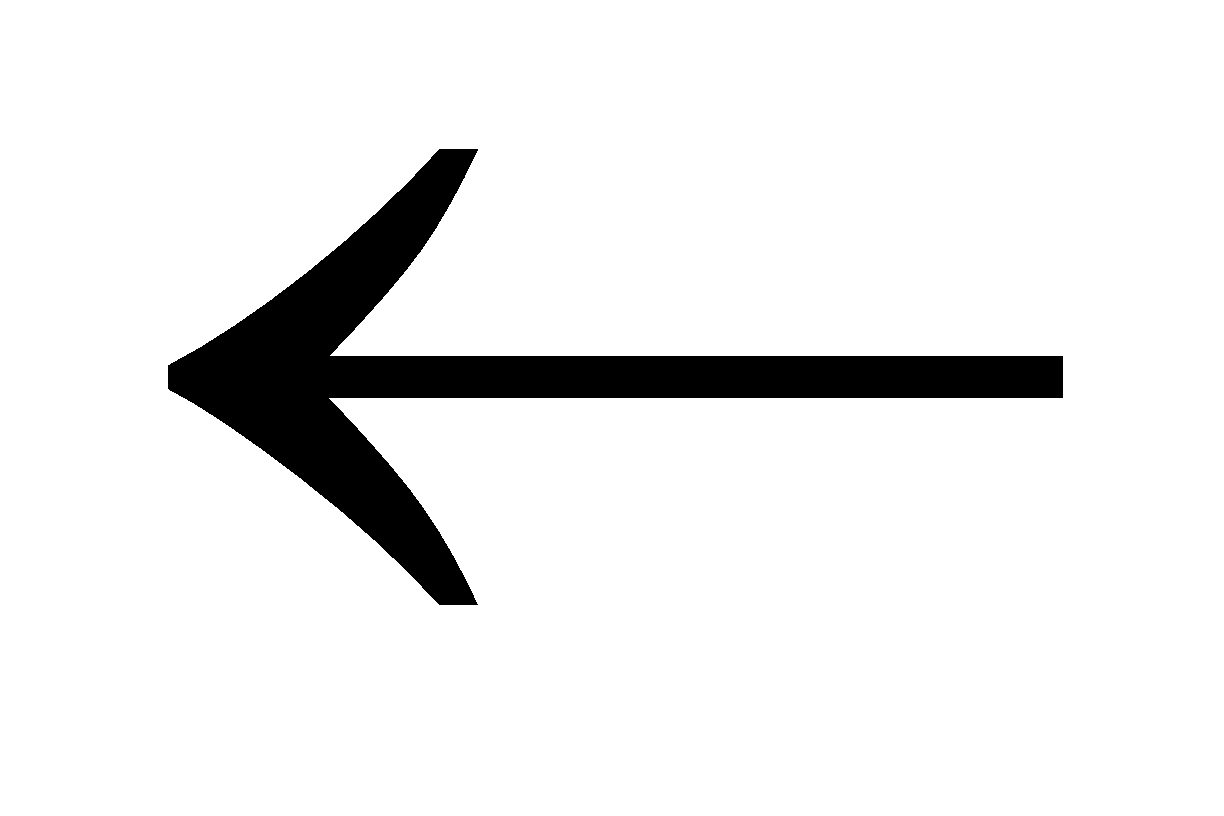_ |
| 7: **Do until** reach goalPoint __ until S is a equal goalpoint |
| 8: Observe current state S |
| 9: if epsilon> epsilon_min: __ Adaptive epsilon |
| 10: epsilon*=epsilon_decay |
| 11: Choose A from S using policy derived from Q |
| 12: **if** random>epsilon: |
| 13: Select a random A |
| 14: **else:** |
| 15: Select max_value from A |
| 16: Take action A, observe R, S’ |
| 17: Q(S,A) __ Q(S,A) + learning_rate[R + discount_factor(maxQ(S’,a) -Q(S,A)] __ Update Q table |
| 18: S__S’ |
| 19: **end for** |
| 19: **return** optimal path |
